# Early Hydration Kinetics of Shell Ash-Based Cementitious Materials: A Low-Field Nuclear Magnetic Resonance Study

**DOI:** 10.3390/ma18143253

**Published:** 2025-07-10

**Authors:** Chuan Tong, Liyuan Wang, Kun Wang, Jianxin Fu

**Affiliations:** 1 School of Civil Engineering, University of Science and Technology Beijing, Beijing 100083, China; tongchuan@sd-gold.com (C.T.); liyuan0421@126.com (L.W.); woken-up@outlook.com (K.W.); 2 Shandong Gold Group (Laizhou) Co., Ltd., Jiaojia Gold Mine, Yantai 261441, China; 3 Department of Environmental and Materials Engineering, Yantai Golden Vocational College, Yantai 261441, China

**Keywords:** shell ash, LF-NMR, K-D model, hydration

## Abstract

This study systematically investigates the effects of shell ash (SA) content (0–10%) on early moisture evolution, pore structure, and hydration kinetics in cement paste using LF-NMR and NG-I-D hydration kinetic models. Key findings include the following: (1) Increased SA content significantly alters moisture phase distribution. Low contents (≤8%) consume free water through rapid CaO hydration, promoting C-S-H gel densification. However, 10% SA causes reduced moisture in 0.16–0.4 μm gel micropores (due to hindered ion diffusion) and abrupt increases in 0.63–2.5 μm pores. (2) Porosity first decreases then increases with SA content, reaching minimum values at 3–5% and 8%, respectively. The 10% content induces abnormal porosity growth from localized over-densification following polynomial fitting (R^2^ = 0.966). (3) Krstulovic–Dabic model analysis reveals three consecutive hydration stages: nucleation–growth (NG), phase boundary reaction (I), and diffusion control (D). The NG stage shows the most intense reactions, while the D stage dominates (>60% contribution), with high model fitting accuracy (R^2^ > 0.9). (4) SA delays nucleation/crystal growth, inducing needle-like crystals at 3% content. Mechanical properties exhibit quadratic relationships with SA content, achieving peak compressive strength (18.6% increase vs. control) at 5% SA. This research elucidates SA content thresholds governing hydration kinetics and microstructure evolution, providing theoretical support for low-carbon cementitious material design.

## 1. Introduction

The global construction industry faces pressing challenges in carbon emission reduction. Cement production, the predominant binder material, emits approximately 0.6–0.9 tons of CO_2_ per ton, contributing 8–10% of global carbon emissions [1,2,3]. Concurrently, over 10 million tons of marine shell waste are generated annually worldwide, with Southeast Asia alone accounting for >30%. The massive accumulation of shells causes land occupation, pathogen proliferation, and coastal ecological pollution. Transforming such biomass waste into high-value construction materials has become crucial for achieving “dual carbon” goals and circular economy synergy [4,5,6]. Industrial solid waste recycling emerges as a key pathway, where marine shell waste demonstrates potential as supplementary cementitious material due to its high CaCO_3_ content (>90%) and reactive CaO generated through calcination [7,8,9].

Utilizing solid waste as cement alternatives presents an economical and eco-friendly solution. Chelluri et al. [10,11] developed cement pastes using slag, fly ash, and rice husk ash substitutes. Hou et al. [12] explored fly ash conversion into C-S-H for homogeneous distribution in cement. Wang et al. [13,14] evaluated ternary solid waste systems (red mud/desulfurized gypsum/fly ash) for cementitious material performance. Wan et al. [15] formulated a green binder from desulfurized gypsum and fly ash, identifying optimal strength at equal FA/cement ratios. Zafar et al. [16] demonstrated fly ash’s enhancement of concrete erosion resistance. Shell ash (SA) not only reduces cement consumption and CO_2_ emissions but also activates silico-aluminous materials through alkaline components, promoting secondary hydration [17,18]. Chen et al. [18] established SA dosage–compressive strength relationships in backfill materials. Naqi et al. [19] attributed SA’s mechanical improvements to enhanced hydration performance. Ez-zaki et al. [20] designed novel thermal-insulating mortars using glass/shell powder hybrids. Maritinez et al. [21] assessed SA’s feasibility as concrete aggregate. Xuan et al. [22] highlighted SA’s dual role in marine pollution mitigation and CO_2_ sequestration through carbonation. Seo et al. [23] identified an optimal 3% SA content for C-H promotion while noting excessive SA inhibits C-S-H formation. As a cement alternative material, shell ash shows significant advantages in resource sustainability and environmental friendliness. Compared with industrial by-products such as fly ash and desulfurized gypsum, shell ash comes from marine waste (such as oyster shells, scallop shells, etc.), has natural renewable properties, does not require energy consumption for high-temperature calcination, and has a lower carbon footprint.

However, existing research predominantly focuses on SA’s long-term mechanical effects in hardened concrete, lacking a systematic understanding of its mechanisms during early hydration stages (<24 h), particularly regarding microscopic moisture migration and pore evolution dynamics [24,25]. Early hydration critically determines initial pore structure, setting time, and durability foundations [12,26], yet conventional techniques (CT/XRD/SEM) face limitations in spatiotemporal resolution and destructive sampling [27], hindering real-time hydration monitoring [28,29,30]. Low-field nuclear magnetic resonance (LF-NMR) technology [31] enables non-destructive, in situ hydration analysis through transverse relaxation time (T_2_) distributions, distinguishing free water (long T_2_, macropores), interlayer water (medium T_2_, mesopores), and gel water (short T_2_, micropores) [32,33]. Deng et al. [34] analyzed the effects of waste glass powder and slag on hydration, compressive strength, and setting time of Portland cement by NMR. Liu et al. [35] proposed a method to analyze the degree of cement hydration based on low-field nuclear magnetic technology and compared it with the results of a thermogravimetric test.

Inverse algorithms further quantify pore distribution functions and connectivity [36,37]. Nevertheless, LF-NMR studies on SA–cement systems remain scarce, particularly regarding SA dosage effects on free water consumption, C-S-H nucleation, and pore fractal characteristics during early hydration [38,39]. SA’s highly reactive CaO may induce localized pH spikes and ion gradients affecting C-S-H nucleation sites and growth kinetics [40], yet the manifestation through moisture redistribution and pore evolution remains unclear [41,42].

This study innovatively combines low-field nuclear magnetic resonance technology with the Krstulovic–Dabic hydration kinetic model, revealing the dynamic coupling law of shell ash dosage (0–10%) on the moisture phase state, pore evolution, and reaction kinetics during the early hydration of gelled materials. By constructing a three-peak analytical quantitative model of the T_2_ spectrum, the dynamic distribution visualization of free water, interlayer water, and gel water in the shell ash–cement system was realized for the first time, breaking through the limitations of the characterization of the microscopic hydrated phase states by traditional testing methods.

The research results not only clarify the optimal dosage interval (5–8%) of shell ash-based gelling materials and their pore control mechanism but also build a cross-scale analysis framework that integrates macro performance and microkinetics, providing theoretical support and method innovation for the resource utilization of industrial solid waste and the design of low-carbon building materials.

## 2. Materials and Methods

### 2.1. Materials

**Figure 1:**
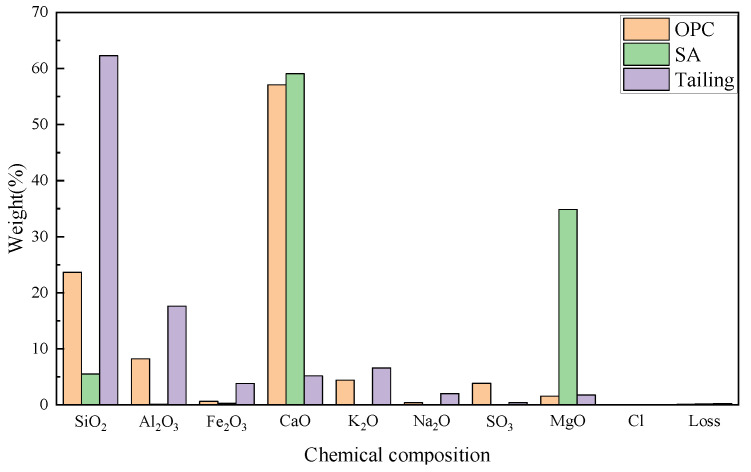
Chemical elements of materials.

**Figure 2:**
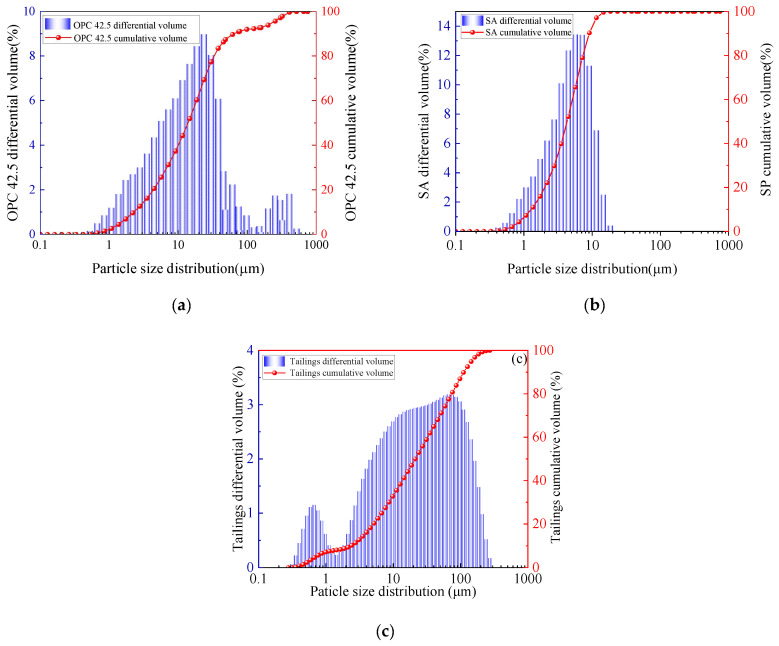
Particle size distribution analysis: (**a**) ordinary Portland cement; (**b**) shell ash; (**c**) tailings.

**Figure 3:**
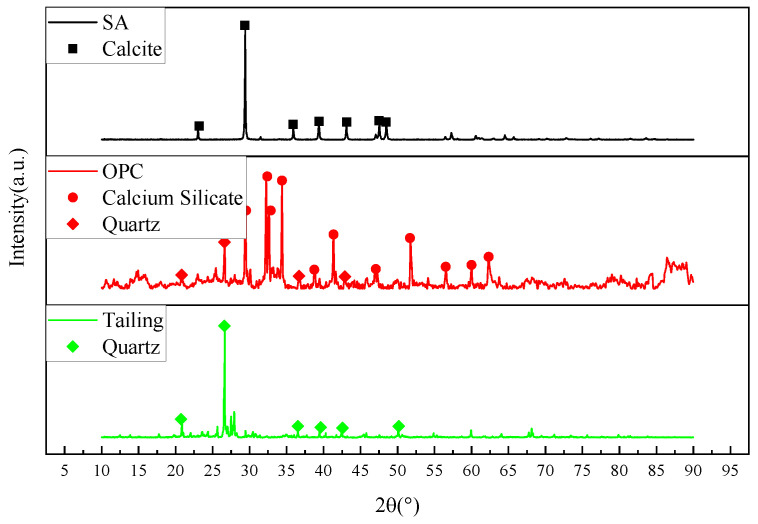
XRD patterns of raw materials.

This study utilizes locally available waste materials, including shell ash (SA) from coastal Yantai and tailings from a gold mine. Ordinary Portland cement 42.5 (OPC) was sourced locally, with laboratory tap water used for mixing. The elemental compositions of shell ash, tailings, and cement were analyzed by XRF, as shown in Figure 1. Particle size distributions obtained through laser diffraction analysis are presented in Figure 2. XRD patterns in Figure 3 reveal the chemical composition of materials.

Shell ash, primarily composed of CaO and MgO with trace Al_2_O_3_ and Fe_2_O_3_, exhibits calcite-type CaCO_3_ (partially calcined to CaO) and magnesite (MgCO_3_) crystalline phases. Gold mine tailings contain 62.27 wt% SiO_2_, while ordinary Portland cement (OPC, specific surface area: 340 m^2^/g, density: 3.15 g/cm^3^) is dominated by CaO (57.1 wt%) and SiO_2_ (23.65 wt%). The study of shell ash in cement hydration is critical due to its triple synergy: (1) Chemical activation—CaO enhances early pozzolanic reactions (C-S-H gel formation), while MgO delays hydration to reduce shrinkage; (2) Sustainable valorization—repurposing coastal shell waste (>10 million tons/year) reduces limestone consumption and landfill pressure; (3) Performance enhancement—its porous structure promotes C-S-H nucleation, and residual calcite improves carbonation resistance. Combined with SiO_2_-rich tailings, shell ash optimizes the CaO/SiO_2_ ratio, balancing environmental benefits (waste recycling) with technical merits (accelerated strength development and durability), positioning it as a high-potential SCM for low-carbon cement systems.

### 2.2. Sample Preparation

To evaluate shell ash’s performance in backfill slurry, a multi-level hydration study was conducted. Considering potential material deterioration from MgO in shell ash, selected substitution rates (3%, 5%, 8%, 10%) of cement with shell ash were tested. Hydration characteristics were analyzed through moisture state evolution in the slurry system:

Gel water: Confined in nano-pores of C-S-H gel. Interlayer water: Trapped between C-S-H gel lamellae or tailings particle gaps. Free water: Unbound water in macropores or slurry surface, remaining non-reactive.

**Figure 4:**
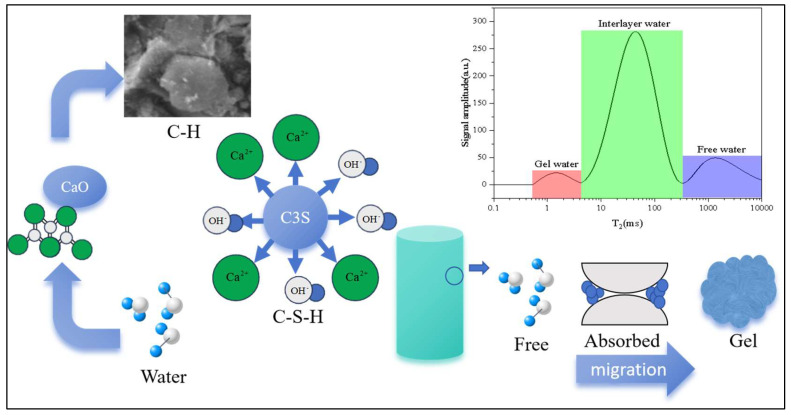
Moisture migration diagram in early curing age.

As illustrated in Figure 4, the experimental approach investigates shell ash’s hydration kinetics by tracking its impacts on these three water phases. The study specifically monitors the following:

Temporal redistribution patterns among water phases; the correlation between CaO/MgO dissolution rates and gel water formation; and threshold content where MgO-induced expansion counteracts densification benefits. This phased water analysis enables precise identification of shell ash’s dual role as both hydration accelerator (through reactive CaO) and potential durability risk factor (via MgO delayed reaction).

Sample preparation procedure: Weigh materials in specified proportions, thoroughly mix in a beaker, then add water. After 5 min of additional stirring, transfer the mixture into a 20 mL glass vial. Detailed mixing ratios are as follows (Table 1):

### 2.3. Experimental Methods

#### 2.3.1. Low-Field NMR

The MesoMR23060H-1 LF-NMR analyzer (Niumag, Suzhou, China) was employed, with a permanent magnet field strength of 0.50 ± 0.08 T, main frequency of 21.3 MHz, and magnet temperature maintained at 32 °C. Prior to sampling, the CPMG pulse sequence was applied with an echo time of 0.1 ms, 15,000 echoes, and 16 scan repetitions. Relaxation signals were analyzed using the built-in InvFit inversion software (Nuclear magnetic resonance analysis application software Ver. 1.0) to obtain T2 distribution spectra.

Testing protocol:

Pure cement backfill slurry and cement slurries with shell ash replacements of 3%, 5%, 8%, and 10% were individually loaded into the instrument and subjected to testing.

#### 2.3.2. The Krstulovic–Dabic Kinetic Model

Based on the K-D kinetic paradigm, cement hydration can be delineated into three cardinal mechanisms: crystalline nucleation–growth (NG), interfacial phase reaction (I), and transport-limited diffusion (D). This tripartite kinetic system exhibits temporospatial concurrence, with macroscopic hydration kinetics regulated by the rate-determining phase exhibiting maximal retardation. The progression of hydration extent (α) as a temporal function (t) for individual mechanistic regimes is mathematically formalized through the following governing equations:

(1) $$\mathrm{NG}:{\left[-\ln(1-\alpha )\right]}^{1/n}=K_{1}(t-t_{0})=K_{1}^{\prime }(t-t_{0})$$

(2) $$I:{\left[1-{\left(1-\alpha \right)}^{1/3}\right]}^{1}=K_{2}r^{-1}(t-t_{0})=K_{2}^{\prime }(t-t_{0})$$

(3) $$D:{\left[1-{\left(1-\alpha \right)}^{1/3}\right]}^{2}=K_{3}r^{-2}(t-t_{0})=K_{3}^{\prime }(t-t_{0})$$

where α represents the degree of hydration; $K_{1}(K_{1}^{\prime })$, $K_{2}(K_{2}^{\prime })$, and $K_{3}(K_{3}^{\prime })$ constitute the kinetic coefficients governing three principal hydration regimes; *t*_0_ denotes the temporal demarcation concluding the dormant phase; n characterizes the crystallographic growth morphology index (with n = 1 corresponding to acicular propagation, n = 2 to lamellar development, and n = 3 to equidimensional expansion); *r* defines the reactive granular dimension participating in the mechanistic sequence.

Through rigorous application of the constitutive kinetic framework, the kinetic flux densities governing the NG, I, and D mechanistic domains become mathematically derivable. The fundamental governing expressions are formulated as follows:

(4) $$\mathrm{NG}:d\alpha /dt=F_{1}(\alpha )=K_{1}^{\prime }n(1-\alpha ){\left[-\ln(1-\alpha )\right]}^{(n-1)/n}$$

(5) $$I:d\alpha /dt=F_{2}(\alpha )=K_{2}^{\prime }\cdot 3{\left(1-\alpha \right)}^{2/3}$$

(6) $$D:d\alpha /dt=F_{3}(\alpha )=K_{3}^{\prime }\cdot 3{\left(1-\alpha \right)}^{2/3}/\left[2-2{\left(1-\alpha \right)}^{1/3}\right]$$

where $F_{1}(\alpha )$,$F_{2}(\alpha )$ and $F_{3}(\alpha )$ represent the equations of the processes of NG, I, and D, respectively.

Through rigorous implementation of the K-D kinetic paradigm, critical reaction descriptors become quantifiable via experimental hydration datasets, enabling fundamental mechanistic elucidation of cementitious matrix activation dynamics.

## 3. Results and Analysis

### 3.1. Analysis of Early Moisture Changes in Cement Slurry

#### 3.1.1. Water Distribution

**Figure 5:**
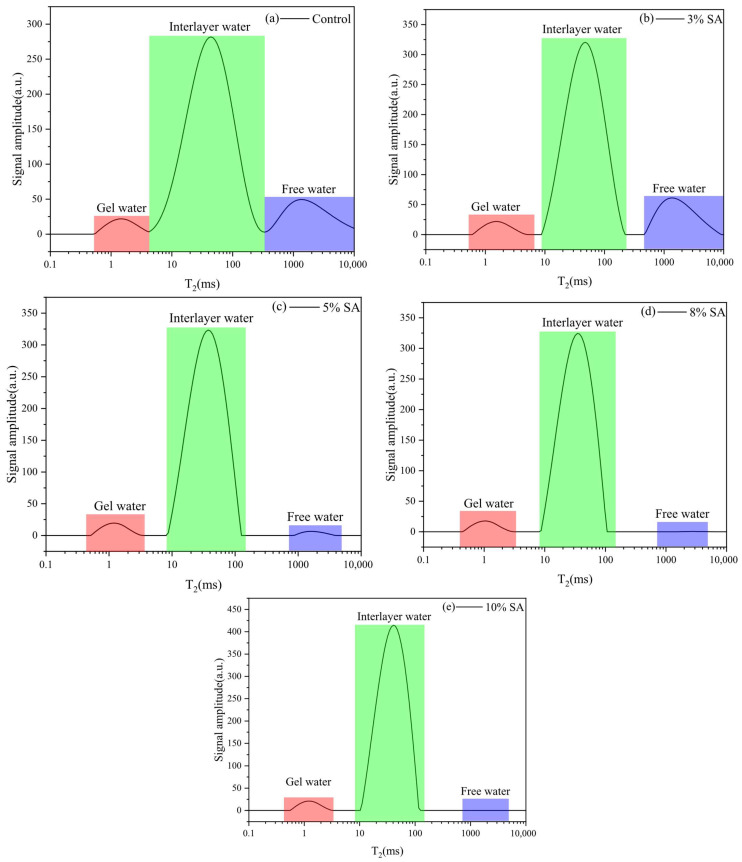
T_2_ relaxation time spectrum from LF-NMR under different SA content.

As LF-NMR uses water molecules as probes, it directly reflects moisture variations during hydration after incorporating shell ash. As shown in Figure 5, the internal changes of cement paste after 10 min of hydration under different shell ash contents are illustrated. The spectra distinctly exhibit three peaks: the first corresponds to gel water, the second to interlayer water, and the third to free water. The peak boundaries are defined at 3–4 ms and 200–300 ms. With 3% shell ash addition, free water content slightly increased but subsequently declined as shell ash content rose. The consumption of moisture by calcium oxide (forming C–H) significantly reduced free water content, accompanied by a gradual rightward shift in its distribution. Interlayer water content notably increased with higher shell ash content, and its T2 distribution became more concentrated. Gel water peaks exhibited minimal changes but shifted slightly leftward, indicating denser gel structures in hydration products due to shell ash addition.

**Figure 6:**
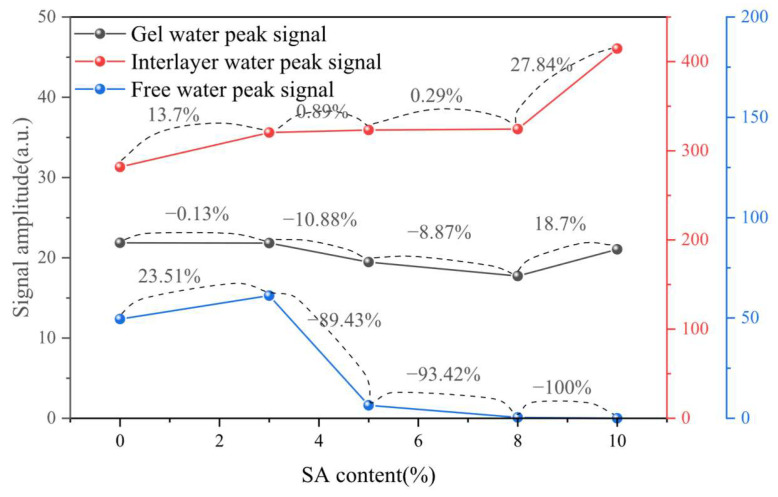
Peak signal amplitude of T_2_ spectrum under different SA content.

From the experimental results of Figure 6, it can be seen that the amount of shell ash has a significant nonlinear regulatory effect on the early moisture state and hydration process of cement slurry:

Gel water: When 3%, 5%, and 8% shell ash were incorporated, the peak intensity of the gel water decreased by 0.13%, 10.88, and 8.87%, respectively, indicating that shell ash may inhibit the formation of some gel water through adsorption or competition reactions under medium and low contents, but the peak intensity increased by 18.7% against the trend at 10%, suggesting that the Ca^2+^ and OH^−^ provided by shell ash at high contents may accelerate the dissolution of silicate minerals and promote the formation of more C-S-H gels.

Interlayer water: At 3% content, the peak intensity of the water signal between layers increased significantly by 13.7%, and at 5% and 8% content, it increased slightly by 0.89% and 0.29%, while at 10% content, it increased by 27.84%. This phenomenon may be due to the low-dose shell ash (3%) optimizing the pore structure through physical adsorption to enhance the water stability between layers, while at high-dose (10%), the slow decomposition of MgCO_3_ continues to release Mg^2+^, which is replaced with the C-S-H interlayer ions, resulting in a significant increase in the interlayer water content.

Free water: The peak intensity of free water increased by 23.51% at 3% content, reflecting that the initial water absorption effect of shell ash is weaker than its accelerated effect on hydration; however, when the content increases to 5%, the peak intensity of free water dropped sharply by 89.43% and completely disappeared at higher contents (8% and 10%), indicating that shell ash undergoes rapid hydration reaction of the porous structure and the active ingredient (CaO/MgO) at higher contents, significantly accelerating the consumption and conversion of free water.

#### 3.1.2. Moisture Ratio

**Figure 7:**
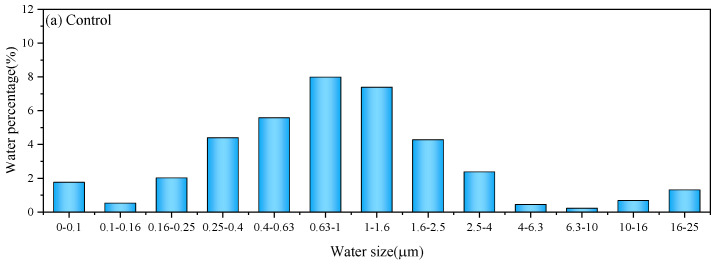

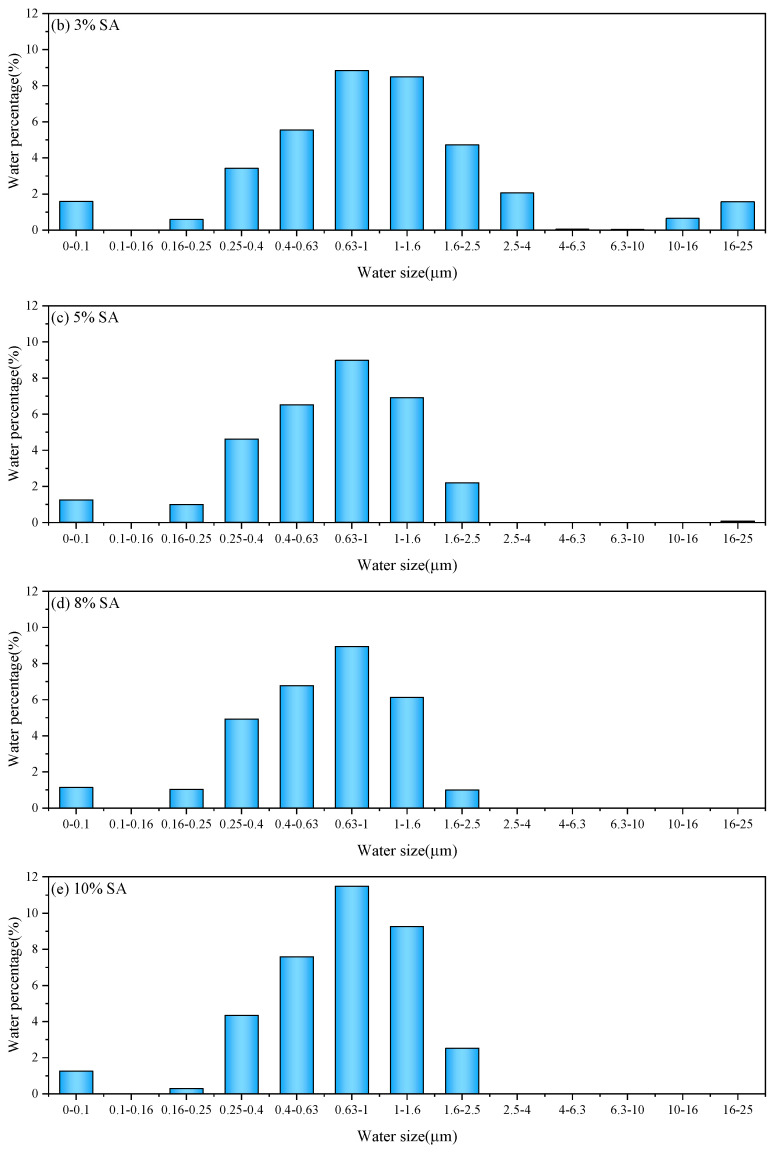
Water volume distribution in cement slurry under different SA content.

Through systematic optimization of interfacial relaxation coefficients and incorporation of phase boundary phenomena governing spin-coupling dynamics during LF-NMR spectrometric analysis, the spatial aqueous fractionation across dimensional pore regimes was computationally resolved through advanced signal deconvolution protocols (Figure 7).

In Figure 7a, water distribution analysis revealed that 0.6–1 μm pores (primarily containing interlayer water) dominated across all five cement paste formulations. With 3% shell ash addition, all 4–10 μm pores transitioned to interlayer water, though some 10–25 μm free water remained. At 5% content, this residual free water is fully converted to interlayer and gel water in Figure 7c. Compared to Figure 7b, the 0.16–0.65 μm fraction significantly increased, indicating preferential water occupation in finer pores and gel micropores.

Higher shell ash contents further reduced 1–2.5 μm pore water, while 0–0.25 μm micropore water remained stable, concentrating predominantly in interlayer water. At 10% content, water in 0.16–0.4 μm pores decreased sharply, whereas 0.63–2.5 μm pores exhibited dramatic increases. This reversal suggests excessive shell ash (≥10%) impedes moisture transport in gel products: overconsumption of water by CaO (forming expansive C–H) creates localized incomplete reactions, dilutes cement content (reducing C-S-H gel formation), and compromises pore-filling capacity.

#### 3.1.3. Porosity Analysis

**Figure 8:**
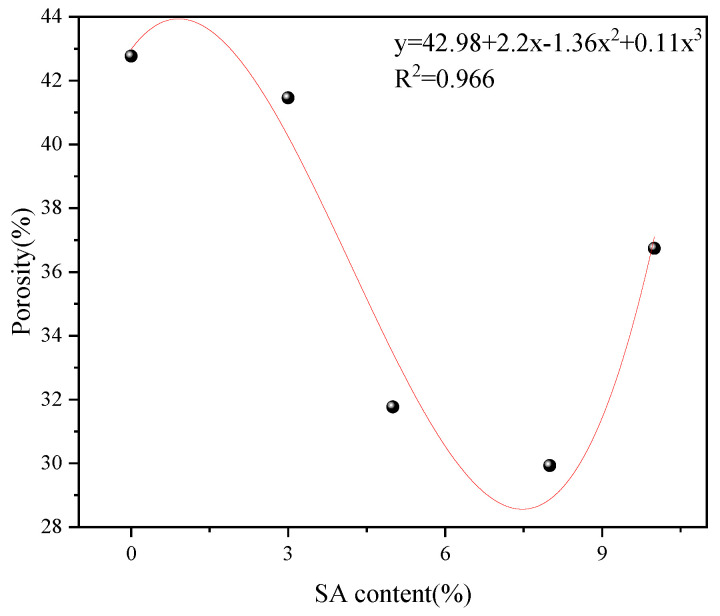
Porosity of cement slurry under different SA content.

Porosity reflects internal moisture variations in cement paste, indicating water consumption during hydration and serving as a critical indicator for evaluating microstructural characteristics and macroscopic performance. By integrating and deriving pore size distribution data from LF-NMR, the porosity of cement paste in 20 mL glass vials after 10 min of hydration was determined (Figure 8). Results show that increasing shell ash content progressively reduced porosity due to enhanced water consumption until a sudden increase occurred at 10% content. The trend followed polynomial fitting (R^2^ = 0.966). The most pronounced porosity decrease occurred between 3% and 5% shell ash additions, with the minimum porosity observed at 8% content.

### 3.2. Analysis of Hydration Dynamics of Shell Ash Based on NMR

#### 3.2.1. Hydration Rate

**Figure 9:**
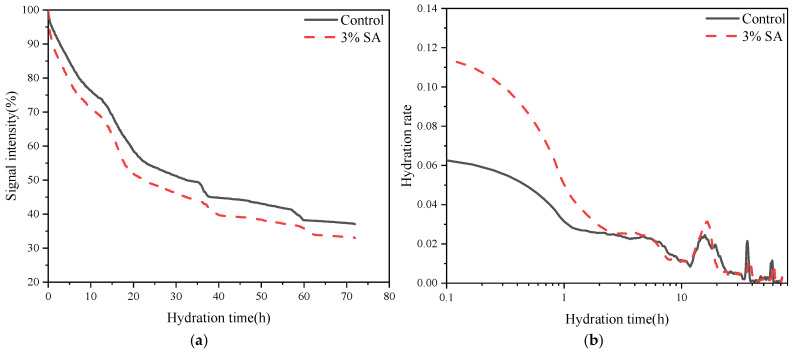
Hydration analysis between the control group and 3% SA group. (**a**) NMR signal intensity. (**b**) Hydration rate.

Continuous variations in water signal amplitude reflect hydration rate throughout the process. By normalizing the initial moisture signal to 100% and monitoring cement paste signals every 5 min over 3 days, hydration differences between mixtures were quantified. Figure 9a displays the amplitude variations and their derivatives (hydration rates) for both 3% shell ash (SA)-modified and pure cement pastes. The control and SA-modified groups exhibited similar signal attenuation (~60% decrease), with residual moisture remaining unconsumed even after 72 h.

Derivative curve analysis revealed the most intense hydration activity within the first 3 h, where the 3% SA paste demonstrated significantly higher hydration rates than the control in Figure 9b. According to experimental data, the hydration activity of the SA paste and control group showed phased differences:

0–3 h: The 3% SA paste demonstrated a significantly higher hydration rate than the control group, indicating stronger hydration activity during the initial phase.

3–4 h: The SA group maintained a relatively high rate but was slightly surpassed by the control group at the 4 h mark, suggesting a threshold for its short-term advantage.

After 10 h: The SA paste exhibited a secondary surge in hydration rate, with acceleration persisting for 6 h before declining. The control group did not show comparable fluctuations during this phase, implying a distinct hydration mechanism specific to SA.

Long-term phase: Beyond the aforementioned surge period, hydration rates in both groups significantly decreased and stabilized. Ultimately, the incorporation of shell ash resulted in an overall hydration rate reduction exceeding 90% after 72 h.

#### 3.2.2. Hydration Kinetic Analysis

Through hydration-responsive spectral parameters, the thermodynamic progression of cementitious hydration states becomes analytically resolvable via multivariate signal regression protocols:

(7) $$\alpha_{t}=\frac{I_{0}-I_{t}}{I_{0}}\cdot \frac{w/c}{\gamma }$$

where *I*_0_ represents the water signal amplitude at the initial hydration stage; It denotes the water signal amplitude at time t; *γ* is the water–cement ratio required for complete cement hydration. Following Powers’ theory, *γ* is assigned as 0.42.

**Figure 10:**
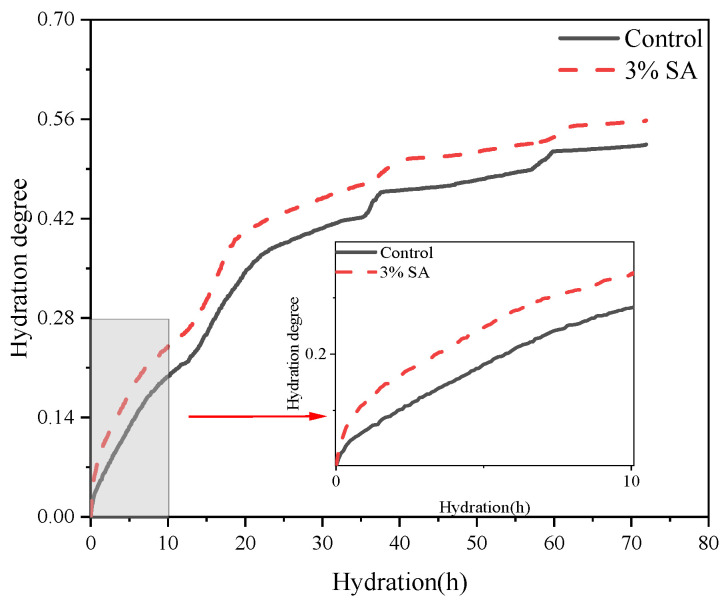
Hydration degree of the control group and 3% SA group.

Using Equation (7), the hydration degree characterized by low-field nuclear magnetic resonance (LF-NMR) was derived, as illustrated in Figure 10. The literature confirms the consistency between LF-NMR-derived hydration degrees and those measured via isothermal calorimetry. This alignment validates the feasibility of establishing the Krstulović–Dabić hydration kinetic model for cementitious materials under theoretical assumptions.

The active ingredients in SA (such as CaCO_3_) accelerate the crystallization of C_3_S (tricalcium silicate) hydration products by providing nucleation sites and its porous structure promotes water diffusion, resulting in a significantly higher hydration rate in the first 3 h than in the control group; at 3–4 h, the local ion concentration saturation or hydration products cover the surface of the particles, and the rate is temporarily limited; after 10 h, the sustained-release alkaline substances or incompletely decomposed organic components in shell ash trigger a secondary reaction, triggering a surge in secondary rates for 6 h, manifested as the local nucleation and growth of new hydration products (such as ettringite) on the microscopic.

**Figure 11:**
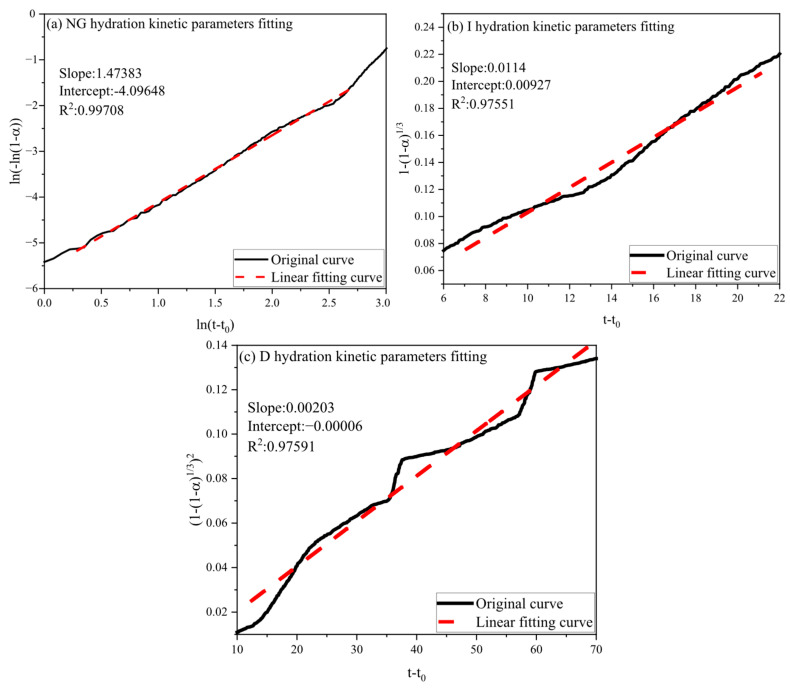
Linear fitting curve for the kinetic parameters of the NG, I, and D processes of the control.

The hydration degree findings from Equation (7) were integrated into Equations (1)–(3) for linear regression analysis. Using the control group as a case, Figure 11 shows the fitting results for NG, I, and D, revealing relatively minor fitting errors. From the slopes and intercepts of the linear regression, specific hydration kinetic parameters were determined: n: 1.47383, K_1_′: 0.062071, K_2_′: 0.0114, and K_3_′: 0.00203, as summarized in Table 2.

**Figure 12:**
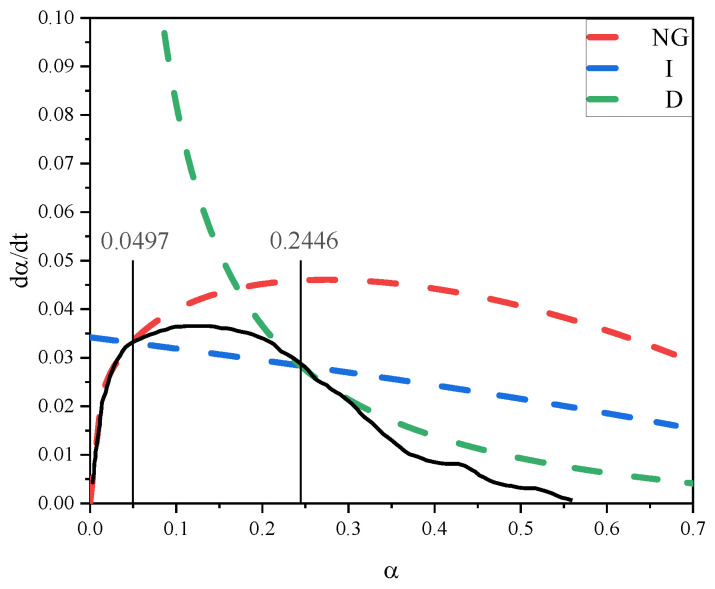
Hydration reaction rate fitting curve of the NG-I-D process of the control.

The appropriate hydration progressions of NG, I, and D were displayed after replacing the kinetic parameters into Equations (4)–(6). Figure 12 depicts the segmentation of the NG-I-D hydration process using LF-NMR. The theoretical curves for NG, I, and D exhibit strong fitting performance. The hydration degree growth rate initially increases and then decreases over time. The hydration process in the control sample progressed from initial nucleation/crystalline development (NG) through interface-controlled reactions (I), ultimately reaching diffusion-dominated stages (D). Within hydration degrees ranging from 0.0497 to 0.2446, the reaction kinetics are predominantly controlled by the phase boundary reaction mechanism (I), whereas diffusion processes prevail as the principal governing factor when hydration advancement exceeds 0.2446.

**Figure 13:**
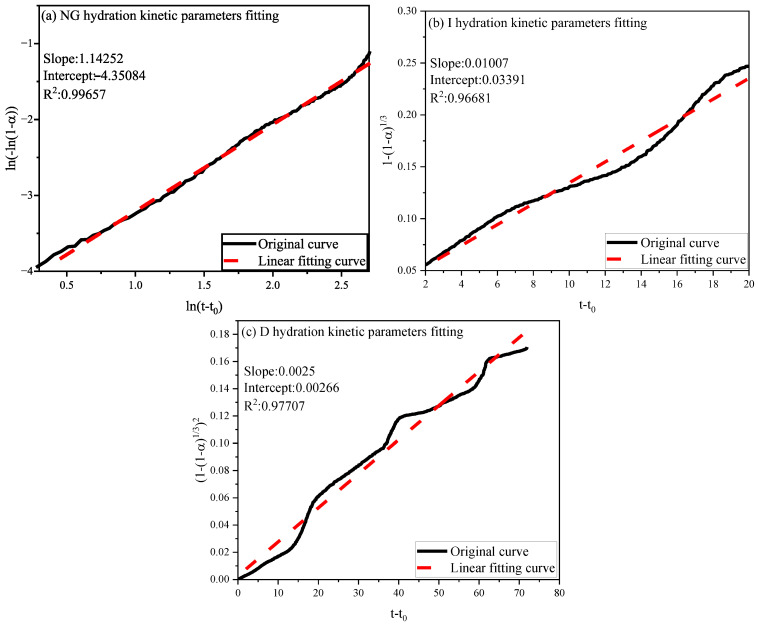
Linear fitting curve for the kinetic parameters of the NG, I, and D processes of 3% SA.

**Figure 14:**
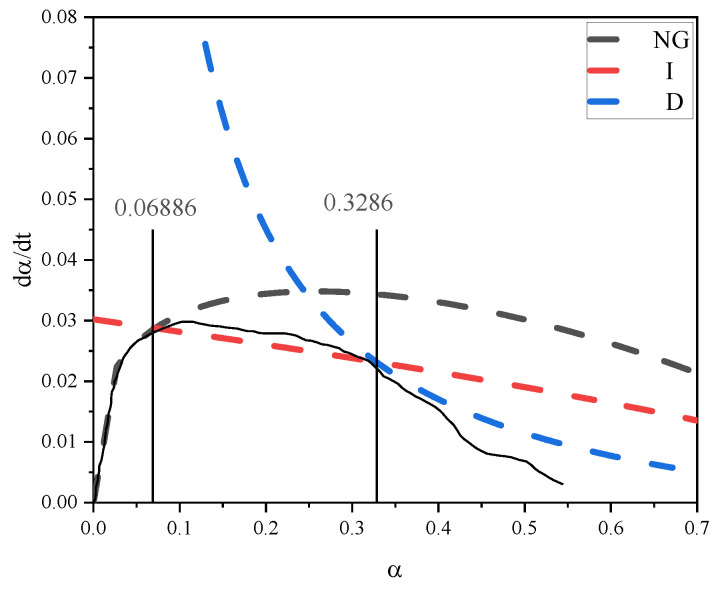
Hydration reaction rate curve of the NG-I-D process of the 3% SA.

Analogous modeling outcomes for the nucleation–growth (NG), interfacial reaction (I), and diffusional (D) stages in the 3% SA composite were derived through computational solutions of Equations (1)–(3), with graphical representations provided in Figure 13. The reaction parameters obtained (n = 1.4252, K_1_′ = 0.047227, K_2_′ = 0.01007, K_3_′ = 0.0025, Table 2) were subsequently implemented in Equations (4)–(6) to mathematically reconstruct the temporal evolution of each mechanistic stage. Figure 14 demonstrates the LF-NMR-assisted phase demarcation within the NG-I-D sequence, revealing accelerated hydration kinetics during the initial 10 h period for both specimens.

The effect of shell ash on the hydration kinetics of cement paste is quantified through kinetic parameters and the evolution of mechanistic stages. By extending the duration of the nucleation–growth (NG) stage (until reaching a hydration degree of α = 0.06886) and enhancing the reaction intensity in this stage (K_1_′ = 0.047227), it is suggested that the active components in shell ash preferentially provide nucleation sites during the initial phase, promoting crystal growth of hydration products. Additionally, its porous structure may enhance local mass transfer efficiency. Subsequently, the interfacial reaction (I) stage (K_2_′ = 0.01007) dominates until α = 0.3286, where the reaction rate is controlled by surface chemical reactions. Shell ash may optimize the reaction pathway through ion exchange or chemical adsorption at particle interface micro-regions. The lower rate constant in the final diffusion-controlled (D) stage (K_3_′ = 0.0025) indicates that shell ash significantly inhibits ion migration in later phases through physical barrier effects, such as the formation of dense product layers or coarsening of pore structures. The crystal growth index (n = 1.4252), intermediate between needle-like (n = 1) and plate-like (n = 2) morphologies, with a stronger tendency toward needle-like growth in the shell ash system, suggests that its surface modification effects may directionally guide the micro-morphology of hydration products, thereby influencing the kinetic pathways of the hydration process. Cross-analysis of kinetic stages further reveals that shell ash accelerates the overall hydration process in early stages by strengthening the synergistic interaction between the NG and I stages (compared to the pure cement system), while diffusion-limiting effects dominate the rate in later phases.

### 3.3. Effect of SA on the Early Strength

#### Analysis of Stress–Strain Curves

**Figure 15:**
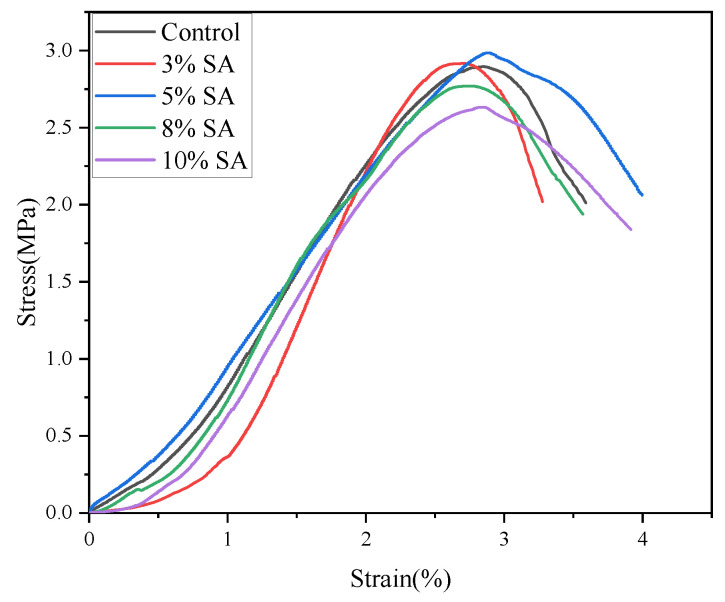
Stress–strain curves of backfill under different SA content at 3 d curing age.

To investigate the mechanical implications of shell ash’s influence on moisture distribution in cement pastes, cylindrical specimens (50 mm diameter × 100 mm height) of the control group and tailings-based mixtures were prepared. These specimens were cured at 22 °C and 90% relative humidity for 3 days before mechanical testing. Figure 15 displays the stress–strain curves of backfill materials with varying shell ash contents. The strength evolution aligns with moisture migration characteristics: increased shell ash content promotes the conversion of moisture into interlayer and gel water, enhancing hydration and strength. Excessive shell ash, however, reduces gel water content and weakens strength. At 5% shell ash content, the backfill achieves peak strength and maximum elastic modulus.

**Figure 16:**
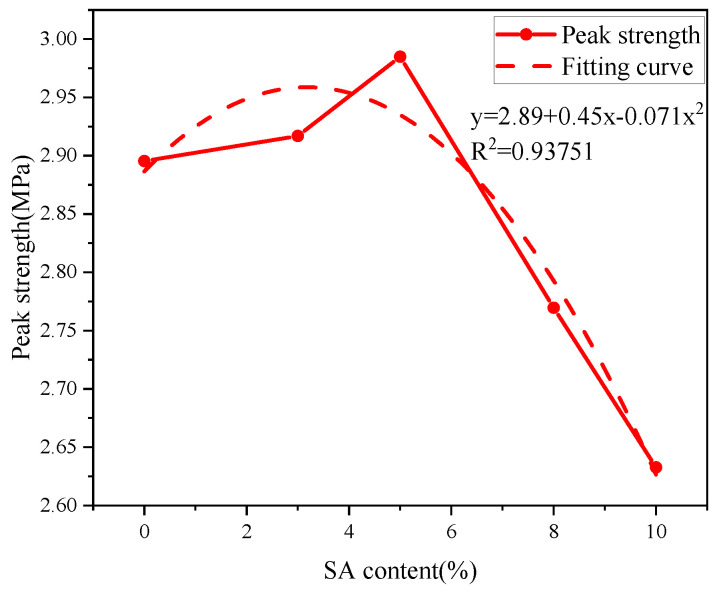
Peak strength vs. SA content of backfill.

A polynomial fit between shell ash content and backfill strength (Figure 16) demonstrates an excellent correlation (R^2^ = 0.93751), with the fitted curve expressed as follows:

(8) $$y=2.89+0.45x-0.071x^{2}$$

The incorporation of shell ash resulted in minimal strength variations in the backfill material. Hydration kinetic curves reveal that its highly reactive calcium oxide reacts with water, forming C-H (calcium hydroxide) during the early stage, which accelerates hydration and mitigates the adverse effects of reduced C-S-H gel formation caused by lower cement content.

### 3.4. Multi-Scale Regulation Mechanism of Shell Ash Content on Hydration Dynamics and Microstructure Evolution

The incorporation of shell ash regulates the early hydration process of cement systems through a multi-scale mechanism. The rapid hydration of high-active CaO in shell ash causes severe consumption of free water, and the released Ca^2+^ and OH^−^ form a local supersaturated environment in the liquid phase, accelerating the nucleation and growth of the C-S-H gel. This process leads to a significant increase in the proportion of moisture in the micropores of 0.16–0.4 μm gel, and the porosity continues to decrease at 3–8% content through the filling effect. However, when the doping amount reaches 10%, the excessive hydration of CaO leads to an imbalance of ion diffusion and the local pH gradient, resulting in non-uniform growth of the C-S-H gel network and abnormal accumulation of pore moisture in 0.63–2.5 μm, revealing the transition from orderly densification to disorderly loosening when the shell ash doping amount exceeds the critical threshold.

Hydration kinetic analysis reveals deep intervention of shell ash on the reaction pathway. Although the reaction rate constant K1’ in the NG stage (nucleation and crystal growth) decreased by 12.6% with the increase in doping, it prolonged the nucleation induction period and promoted the shift of the crystal growth index n to the needle-like morphology (1.425 → 1.474), indicating that shell ash changes the crystallization habits of C-S-H by regulating the solution chemical environment. This transformation of crystal morphology forms a coupling effect with the evolution of pore structure: the cross-growth of needle-shaped C-S-H strengthens the mechanical interlock of the gel network, but the contribution rate of stage D (diffusion control) exceeds 60% under excessive doping, indicating that the deterioration of pore connectivity ultimately dominates the later hydration process, forming the kinetic characteristics of “pre-stage acceleration-late suppression”. This is consistent with the macro conclusion in reference [7]: the use of shells as cement substitutes can shorten the setting time of concrete, reduce workability, and increase density due to the curing age.

Compared with reference [17], it is found that the evolution of mechanical properties comprehensively reflects the above-mentioned multi-factor synergy. When the shell ash content amount is 5%, the optimal hydration degree of CaO achieves efficient conversion of free water, and the porosity is reduced to a minimum (23.8% lower than the benchmark group). At the same time, the hydration degree threshold α1 in the NG-I stage is increased by 38.6%, which prompts the C-S-H gel to form a dense three-dimensional network structure, corresponding to the peak compressive strength increase by 18.6%. However, after the doping amount exceeds 8%, the pore coarsening caused by local overdense and the obstruction of diffusion in stage D form positive feedback, which makes the intensity–doption curve show a significant nonlinear attenuation, confirming the threshold regulation effect of shell ash doping amount on the microstructure–macross performance correlation.

## 4. Conclusions

This study investigated shell ash-modified backfill materials using LF-NMR technology and hydration kinetic modeling, yielding four key findings:

(1) Elevated shell ash content increases interlayer water while reducing free water distribution with stable gel water levels. At ≤8% content, shell ash enhances C-S-H gel densification through CaO hydration. The 0.6–1 μm pore fluid dominated across mixtures, though 10% shell ash increased 0.63–2.5 μm moisture fractions while restricting transport in gel products.

(2) Porosity decreased polynomially with rising shell ash content until an abrupt increase at 10% content. The maximum reduction occurred between 3–5% additions, reaching minimal porosity at 8% content.

(3) The K-D model effectively characterized shell ash–cement hydration kinetics, showing sequential NG-I-D stage transitions. NG demonstrated the highest reaction intensity, while D dominated hydration duration.

(4) SA extended NG regime duration and intensified hydration kinetics. Crystal growth displayed transitional morphology between needle-like and plate-like structures, with 3% content favoring needle-like growth. Mechanical strength showed a polynomial correlation with ash content, peaking at 5% content.

## Figures and Tables

**Table 1:** Experiment design (weight/g).

Name	OPC	Tailing	Water	SA	c/t	w/c
Control	100	400	50		1:4	0.5
3% SA	97			3		
5% SA	95			5		
8% SA	92			8		
10% SA	90			10		

**Table 2:** Kinetic parameters of the NG-I-D process.

Sample	n	K_1_^′^	K_2_^′^	K_3_^′^	Mechanism	α_1_	α_2_
Control	1.47383	0.062071	0.0114	0.00203	NG-I-D	0.0497	0.2446
3% SA	1.4252	0.047227	0.01007	0.0025	NG-I-D	0.06886	0.3286

## Data Availability

The original contributions presented in this study are included in the article. Further inquiries can be directed to the corresponding author.

## References

[1] Huang L., Krigsvoll G., Johansen F., Liu Y., Zhang X. (2018). Carbon emission of global construction sector. Renew. Sustain. Energy Rev..

[2] Fu J.-X., Wang K., Wang J. (2023). Internal pore evolution and early hydration characterization of fly ash cement backfill. J. Build. Eng..

[3] Kusin F.M., Hasan S.N.M.S., Molahid V.L.M., Soomro M.H. (2024). Dual adoption opportunities and prospects for mining and industrial waste recovery through an integrated carbon capture, utilization and storage. Sustain. Prod. Consum..

[4] Wang J., Zhu X., Wang J., Chen Y., Liu Y., Hu Z., Liu J., Maboudian R., Monteiro P.J.M. (2025). Does the hydration process of supplementary cementitious materials affect the aging creep of blended cement paste?. Cem. Concr. Res..

[5] Dahish H.A., Alfawzan M.S., Tayeh B.A., Abusogi M.A., Bakri M. (2023). Effect of inclusion of natural pozzolan and silica fume in cement - based mortars on the compressive strength utilizing artificial neural networks and support vector machine. Case Stud. Constr. Mater..

[6] Wang K., Fu J., Wang J. (2024). The ratio optimization and hydration mechanism of multi source solid waste cementitious materials. Constr. Build. Mater..

[7] Hasan K., Karim A., Islam M.T., Ali M.B., Rahman M.T., Rahman M.M., Yahaya F.M. (2023). A review of the mechanical properties and durability of concrete containing recycled seashells as a partial cement replacement. J. Mater. Cycles Waste Manag..

[8] Li G., Xu X., Chen E., Fan J., Xiong G. (2015). Properties of cement-based bricks with oyster-shells ash. J. Clean. Prod..

[9] Maglad A.M., Mydin M.A.O., Datta S.D., Tayeh B.A. (2023). Assessing the mechanical; durability, thermal and microstructural properties of sea shell ash based lightweight foamed concrete. Constr. Build. Mater..

[10] Chelluri S., Hossiney N. (2024). Performance evaluation of ternary blended geopolymer binders comprising of slag, fly ash and brick kiln rice husk ash. Case Stud. Constr. Mater..

[11] Sithole T., Mashifana T., Mahlangu D., Tchadjié L. (2023). Effect of binary combination of waste gypsum and fly ash to produce building bricks. Sustain. Chem. Pharm..

[12] Hou X., Ma S., Wang X., Ou Y., Liu R. (2024). Transformation of fly ash into a fluffy C-S-H material: Enhancing chemical properties and advancing the utilization of portland cement. Constr. Build. Mater..

[13] Wang Y., Liu X., Zhu X., Zhu W., Yue J. (2023). Synergistic effect of red mud, desulfurized gypsum and fly ash in cementitious materials: Mechanical performances and microstructure. Constr. Build. Mater..

[14] Feng Y., Qi W., Zhao Q., Huang Y., Ren Q., Qi W., Kong F. (2023). Synthesis and characterization of cemented paste backfill: Reuse of multiple solid wastes. J. Clean. Prod..

[15] Wan Y., Hui X., He X., Li J., Xue J., Feng D., Liu X., Wang S. (2022). Performance of green binder developed from flue gas desulfurization gypsum incorporating Portland cement and large-volume fly ash. Constr. Build. Mater..

[16] Zafar I., Alqahtani F.K. (2021). Effectiveness of extended curing for fly ash concrete against corrosion propagation under severe chloride exposure. Struct. Concr..

[17] Li Y., Fu J., Wang K., He Z. (2024). Influence of shell ash on pore structure and mechanical characteristics of cemented tailings backfill. Constr. Build. Mater..

[18] Chen B., Peng L., Zhong H., Zhao Y., Meng T., Zhang B. (2023). Improving the mechanical properties of mussel shell aggregate concrete by aggregate modification and mixture design. Case Stud. Constr. Mater..

[19] Naqi A., Siddique S., Kim H.-K., Jang J.G. (2020). Examining the potential of calcined oyster shell waste as additive in high volume slag cement. Constr. Build. Mater..

[20] Ez-zaki H., El Gharbi B., Diouri A. (2018). Development of eco-friendly mortars incorporating glass and shell powders. Constr. Build. Mater..

[21] Martínez-García C., González-Fonteboa B., Martínez-Abella F., López D.C. (2017). Performance of mussel shell as aggregate in plain concrete. Constr. Build. Mater..

[22] Xuan M.-Y., Cho H.-K., Wang X.-Y. (2023). Performance improvement of waste oyster-shell powder–cement binary system via carbonation curing. J. Build. Eng..

[23] Seo J.H., Park S.M., Yang B.J., Jang J.G. (2019). Calcined Oyster Shell Powder as an Expansive Additive in Cement Mortar. Materials.

[24] Li Z., Tong T., Shi J., Wang Y., Jin P. (2024). Effect of pores and moisture on the mechanical properties of calcium silicate hydrate gels at mesoscale. Constr. Build. Mater..

[25] Hu S., Xu Z., Ma X., Huo Y., Yang Y. (2024). Preparation of C-S-H seeds from solid waste and its application as portland cement accelerator. Constr. Build. Mater..

[26] Morales-Cantero A., De La Torre A.G., Cuesta A., Santacruz I., Bernal I.M.R., Mazanec O., Dalla-Libera A., Borralleras P., Aranda M.A.G. (2024). In situ synchrotron powder diffraction study of LC3 cement activation at very early ages by C-S-H nucleation seeding. Cem. Concr. Res..

[27] Li J., Cao S., Song W. (2023). Distribution development of pore/crack expansion and particle structure of cemented solid-waste composites based on CT and 3D reconstruction techniques. Constr. Build. Mater..

[28] Wang K., Fu J., Zhaobo M., Bangyi Z. (2025). Investigation of frozen rock behavior: Mechanical properties, pore structure, and strength under confining pressures. Cold Reg. Sci. Technol..

[29] Liu Y., Deng H., Jiang Z., Tian G., Wang P., Yu S. (2024). Pore structure characteristics of artificial sand aggregate mortar. J. Build. Eng..

[30] Zhang C., Wang J., Song W., Fu J. (2024). Study on shear behavior and microstructure of rock and cemented paste backfill interface. Constr. Build. Mater..

[31] Liu L., He Z., Cai X., Fu S. (2021). Application of Low-Field NMR to the Pore Structure of Concrete. Appl. Magn. Reson..

[32] Yu J., Wang H., Sun X., Chen Z., Dong W. (2024). Characterizing connectivity and tortuosity of pore network based on LF-NMR method to assess the water permeability of white cement mortar. J. Build. Eng..

[33] Zhang C., Song W., Taheri A., Fu J., Zhao T., Tan Y. (2024). Hydration mechanism and mechanical-thermal correlation of cemented paste backfill under different curing temperatures. J. Build. Eng..

[34] Deng X., Tan H., Yang Z., He X., Wang J., Jian S., Huang J., Yang J. (2024). C-S-H based nucleation seed prepared from waste glass powder and carbide slag and its application in portland cement. Constr. Build. Mater..

[35] Liu H., Sun Z., Yang J., Ji Y. (2021). A novel method for semi-quantitative analysis of hydration degree of cement by 1H low-field NMR. Cem. Concr. Res..

[36] Liu S., Huang Z. (2023). Exploration of microstructure characteristics and mechanical behaviors of thermal-damaged argillaceous sandstone via LF-NMR and µ-CT technologies. Geomech. Geophys. Geo-Energ. Geo-Resour..

[37] Liu Y., Wu Z., Weng L., Wu L., Xu X., Liu Q. (2023). Experimental Study on the Grouting Diffusion Process in Fractured Sandstone with Flowing Water Based on the Low-Field Nuclear Magnetic Resonance Technique. Rock. Mech. Rock. Eng..

[38] Zhan P., Wang J., Zhao H., Li W., Shah S.P., Xu J. (2024). Impact of synthetic C-S-H seeds on early hydration and pore structure evolution of cement pastes: A study by 1H low-field NMR and path analysis. Cem. Concr. Res..

[39] Qiu B., Fan L., Ma C., Yang Q., Du X. (2023). Effects of ice content on compression characteristics of frozen sandstone by in-situ NMR technology. Geomech. Geophys. Geo-Energ. Geo-Resour..

[40] Sophia M., Sakthieswaran N. (2019). Waste shell powders as valuable bio- filler in gypsum plaster – Efficient waste management technique by effective utilization. J. Clean. Prod..

[41] Jin M., Ma Y., Li W., Huang J., Yan Y., Zeng H., Lu C., Liu J. (2023). Multi-scale investigation on composition-structure of C-(a)-S-H with different al/si ratios under attack of decalcification action. Cem. Concr. Res..

[42] She A., Ma K., Yao W., Zuo J., Liao G. (2022). Hydration kinetics of cementitious materials based on low-field NMR and isothermal calorimetry. Fuller. Nanotub. Carbon Nanostruct..

